# Fusion of the Manubriosternal and Xiphisternal Junctions on Computed Tomography: A Probabilistic and Threshold-Based Approach to Age Assessment

**DOI:** 10.3390/diagnostics16213629

**Published:** 2026-11-03

**Authors:** Omur Sanyuz, Elif Hocaoglu, Marine Dario, Eirini Chasapi, Elena F. Kranioti

**Affiliations:** 1 Council of Forensic Medicine, Ministry of Justice, Istanbul 34196, Türkiye; omurkaymaz@yahoo.com; 2 Department of Radiology, Bakırkoy Dr. Sadi Konuk Training and Research Hospital, Health Sciences University, Istanbul 34000, Türkiye; drelifhocaoglu@hotmail.com; 3 Department of Legal Medicine, CHU Montpellier, University of Montpellier, 34000 Montpellier, France; 4 Forensic Medicine Unit, University of Crete, 71500 Heraklion, Greece; eirinichasape@gmail.com

**Keywords:** sternum, forensic age estimation, adult age estimation, computed tomography, manubriosternal joint, xiphisternal joint, probabilistic approach, likelihood ratio

## Abstract

**Background/Objectives:** Sternal fusion has been studied as an adult age indicator, but findings remain inconsistent, partly because variable fusion processes have been interpreted within deterministic frameworks. We reassessed manubriosternal and xiphisternal fusion on computed tomography (CT) using a probabilistic, threshold-based approach. **Methods:** Thoracic CT examinations of 512 individuals aged 30–65 years were retrospectively analysed. Manubriosternal fusion was classified on a three-stage ordinal scale and xiphisternal fusion dichotomously. Fusion was examined across five age bands (30–40, 40–50, 50–55, 55–60 and 60–65 years) and for age thresholds of 50, 55 and 60 years using sensitivity, specificity and likelihood ratios (LRs). **Results:** Manubriosternal fusion overlapped extensively across age bands, with a significant but weak association with age (Kruskal–Wallis *p* < 0.001; ε^2^ = 0.060). Xiphisternal fusion increased from 37.6% (30–40 years) to 77.3% (55–60 years) and plateaued at 75.7% (60–65 years; Mann–Whitney *p* < 0.001), leaving one quarter of 60–65-year-olds non-fused. Xiphisternal fusion increased the odds of being ≥50, ≥55 and ≥60 years old (odds ratios 3.15, 3.14 and 2.57), but its evidential value was modest (positive LRs 1.38–1.55; negative LRs 0.48–0.54). Adding manubriosternal stage improved discrimination for the ≥55-year threshold in an exploratory model (area under the curve 0.628 to 0.676; *p* = 0.002). **Conclusions:** CT-based sternal fusion is unsuitable for deterministic or standalone age estimation. Xiphisternal fusion provides only modest, supplementary information that may be combined with other indicators when CT is already available for clinical or postmortem purposes; it does not justify CT acquisition for age assessment.

## 1. Introduction

Age estimation in forensic medicine and forensic anthropology constitutes a cornerstone of medico-legal decision-making, with direct implications for criminal responsibility, civil status, social rights, and human identification. While substantial methodological advances have been achieved for children, adolescents, and young adults, reliable age assessment in mature adults remains one of the most persistent and unresolved challenges in the field [1,2,3,4]. Following the completion of growth, skeletal maturation becomes increasingly decoupled from chronological age, resulting in pronounced interindividual biological variability that limits the precision of classical age indicators.

Adult age estimation has traditionally relied on morphological changes observed in skeletal structures such as the pubic symphysis, auricular surface, ribs, and cranial sutures [2,3,4]. Although these methods provide broad age intervals, their discriminatory power decreases markedly in middle and advanced adulthood, where overlap between age ranges becomes substantial. In addition, cranial sutures and the auricular surface are considered less appropriate for forensic casework because of their high error rates. In response to these limitations, secondary ossification sites and synchondroses have been explored as potential indicators of later skeletal maturation, particularly for individuals beyond the third decade of life.

The sternum, composed of the manubrium, mesosternum, and xiphoid process, represents one such structure of interest. Because it is frequently preserved in both postmortem examinations and computed tomography (CT) imaging, the sternum has long attracted interest as a potential source of age-related information in forensic practice. The manubriosternal and xiphisternal junctions are secondary cartilaginous joints that may undergo partial or complete fusion during adulthood. Early anatomical investigations by Dwight [5] and Jit and Bakshi [6], together with subsequent autopsy- and radiology-based studies [7,8,9,10,11,12,13,14,15,16,17,18,19], demonstrated considerable variability in the timing of sternal fusion. Fusion has been described across wide and overlapping age ranges, and in many individuals fusion may be absent even at advanced ages. Consequently, sternal fusion has often been regarded as unreliable or of limited forensic value for adult age estimation [7,8,9,10,11,12,13,14,15,16].

More recently, multidetector and three-dimensional CT studies have renewed interest in the sternum by allowing detailed in vivo assessment of the manubriosternal and xiphisternal junctions. Investigations performed in Japanese, Egyptian, Indian, Mediterranean and other populations have consistently demonstrated statistically significant associations between fusion status and chronological age, while simultaneously reporting substantial overlap between age groups, thereby limiting deterministic age estimation [20,21,22,23,24,25,26,27,28,29,30].

These heterogeneous findings most likely reflect several interacting factors rather than a single source of variation [31,32]. Interindividual biological variability, inter-population differences in skeletal maturation, differences in study populations, and methodological diversity in imaging techniques and staging systems all contribute to the inconsistencies reported in the literature.

Advances in cross-sectional imaging, particularly CT, have substantially improved the assessment of complex skeletal structures. Thin-slice multidetector CT enables detailed visualisation of the sternum, reduces projectional ambiguity inherent to conventional radiography, and allows retrospective analysis of large clinical populations. CT-based approaches have been successfully applied to multiple anatomical regions for forensic age assessment, including the medial clavicular epiphysis, cranial synchondroses, ribs, and pelvic structures [20,21,22,23,24,33,34,35].

Despite these technological advances, the forensic relevance of manubriosternal and xiphisternal fusion assessed by CT remains debatable. Although several studies have demonstrated statistically significant age-related trends, particularly for the xiphisternal junction, the extensive overlap observed between younger and older adults has consistently limited deterministic age estimation. Consequently, some authors have proposed age thresholds or staging systems, whereas others have concluded that sternal fusion has little practical value for forensic age estimation [21,22,23,24,25,26].

We propose that these apparently conflicting conclusions arise not only from genuine biological variability but also from differences in the interpretative framework applied to similar empirical observations. Biological variability determines the observed distributions, whereas the analytical framework influences how these findings are interpreted in a forensic context. When evaluated within a deterministic age-estimation paradigm, overlapping distributions are regarded as evidence of poor discriminatory performance. Conversely, within a probabilistic framework, the same distributions may still provide supplementary information on age likelihood without implying precise chronological age prediction.

The present study revisits manubriosternal and xiphisternal fusion using thin-slice CT in a large adult population, explicitly adopting a probabilistic and threshold-based perspective. Rather than attempting to derive point estimates of chronological age, this work examines how fusion patterns modify age likelihoods across adulthood. We hypothesised that xiphisternal fusion would demonstrate a monotonic probability shift toward older adult age thresholds, whereas manubriosternal fusion stages would remain broadly overlapping and weakly informative. By evaluating sternal fusion as a potential modifier of age likelihood rather than as a deterministic age estimator, this study aims to quantify both its contribution and its limitations in forensic age assessment.

## 2. Materials and Methods

### 2.1. Study Design and Population

This retrospective observational study was based on thoracic CT examinations performed at Bakırköy Dr. Sadi Konuk Training and Research Hospital (Istanbul, Turkey). CT scans acquired between June 2013 and December 2013 for clinical indications unrelated to the sternum—primarily trauma assessment and acute medical evaluation—were retrospectively screened. The study population consisted of adult patients undergoing thoracic CT for non-forensic clinical indications.

Because this was a retrospective observational study, no a priori sample size calculation was performed. Instead, all consecutive CT examinations meeting the predefined inclusion and exclusion criteria during the study period were included.

Individuals aged 30 to 65 years at the time of imaging were considered eligible. This age range was deliberately selected to focus on post-growth adult skeletal maturation while avoiding late adolescent transitional stages and very advanced ages in which degenerative changes may predominate. After application of all exclusion criteria, 512 individuals constituted the final study population. All CT examinations were evaluated by the observers directly from the original imaging data.

Eligible individuals were required to have CT examinations of sufficient image quality to permit evaluation of both the manubriosternal and xiphisternal junctions. Exclusion criteria included acute or healed sternal fractures, congenital chest wall deformities (including pectus excavatum and pectus carinatum), Marfan syndrome, previous sternal surgery, severe motion or reconstruction artefacts, and systemic or neoplastic diseases potentially affecting sternal morphology or treated with corticosteroids, radiotherapy, or chemotherapy. Cases in which ossification of the xiphisternal junction had begun but was incomplete (partial xiphisternal fusion) were also excluded, so that xiphisternal status could be classified unambiguously (Section 2.3.2).

Sex was recorded for all individuals. Exploratory sex-stratified analyses were performed to evaluate potential differences in fusion patterns between males and females. However, because the primary objective of the study was to evaluate the overall probabilistic behaviour of sternal fusion across adulthood rather than to establish sex-specific reference standards, the principal analyses were performed using the combined study population. Sex-stratified descriptive data are presented in Tables S1 and S2 (Supplementary Materials).

The study was approved by the Ethics Committee of Bakırköy Dr. Sadi Konuk Training and Research Hospital, Istanbul (approval No. 2013/123). Owing to the retrospective design and anonymisation of all CT datasets, the requirement for informed consent was waived.

### 2.2. CT Acquisition and Image Reconstruction

All CT examinations were performed using a 40-row multidetector CT scanner (Siemens Medical Solutions, Erlangen, Germany) according to a standard thoracic CT protocol. Scans were acquired with patients in the supine position, arms elevated where clinically feasible. Acquisition parameters were as follows: tube voltage 120 kV, effective tube current 120 mAs, slice thickness 1.0 mm, and reconstruction interval 1.0 mm.

Raw image data were transferred to a dedicated workstation (Leonardo, Siemens), where multiplanar reconstructions (MPR) were generated in sagittal and coronal planes. Sagittal reconstructions were prioritised for evaluation of the sternum, as they allow continuous visualisation of the manubriosternal and xiphisternal junctions along their anatomical axes and minimise partial volume effects. For the figures, panel margins were trimmed to equal widths and the same linear brightness and contrast adjustment was applied uniformly to all images using Python (Pillow, version 12.3); arrows and panel letters were added, and no filtering or other image processing was performed. The unprocessed original images are available from the authors on request.

### 2.3. Evaluation of Sternal Fusion

All assessments were performed on thin-slice sagittal CT images under standardised viewing conditions.

#### 2.3.1. Manubriosternal Junction

Fusion of the manubriosternal junction was assessed visually and classified using a three-stage ordinal system, reflecting increasing degrees of osseous continuity:

- Stage 1 (no fusion): a clearly defined joint space without osseous bridging;
- Stage 2 (partial fusion): incomplete osseous bridging, typically focal or central, with residual joint space;
- Stage 3 (complete fusion): continuous osseous union across the entire junction.

**Figure 1:**
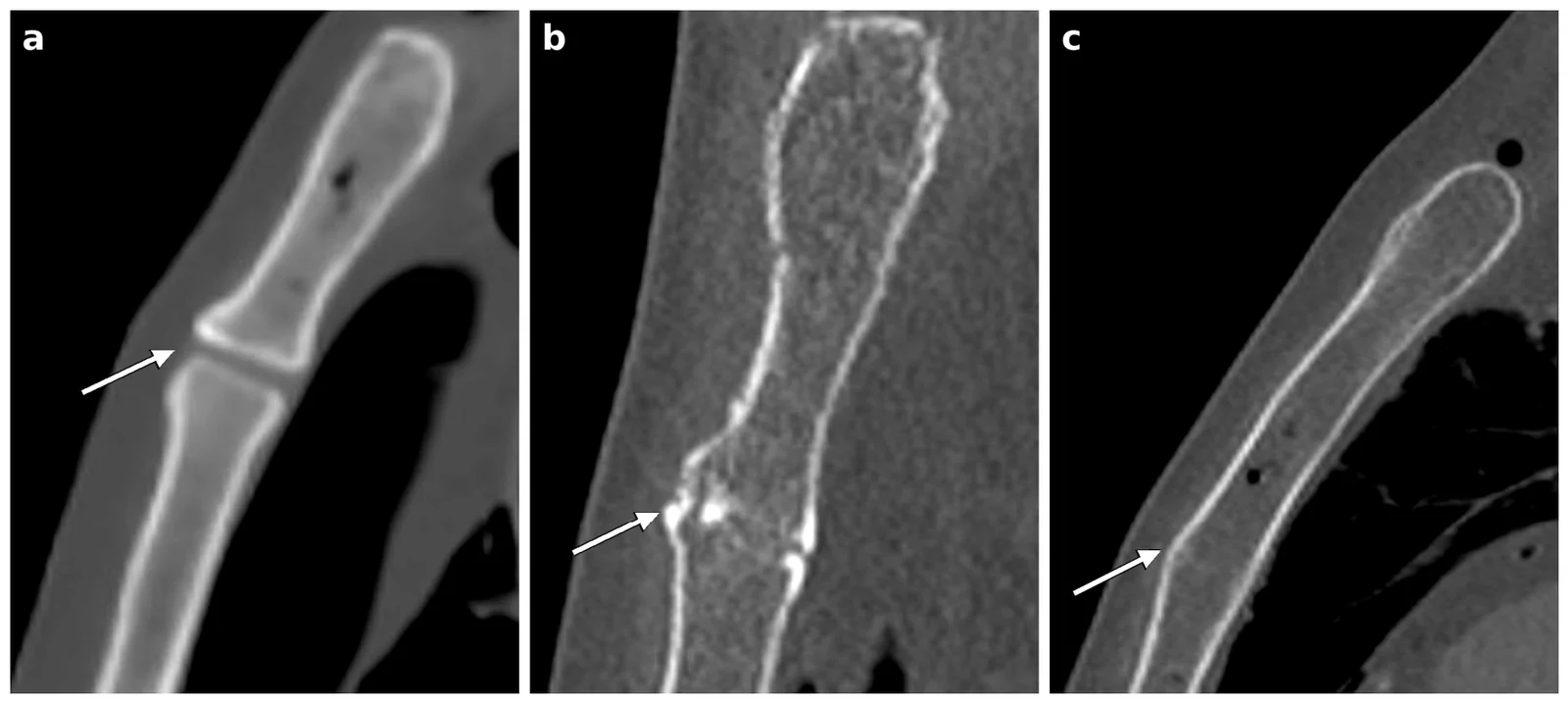
Representative sagittal CT images of the manubriosternal junction. (**a**) Stage 1 (no fusion): clearly defined joint space without osseous bridging. (**b**) Stage 2 (partial fusion): incomplete, irregular osseous bridging with residual joint space. (**c**) Stage 3 (complete fusion): continuous osseous union across the entire junction. Arrows indicate the manubriosternal junction.

This staging system was selected to maintain comparability with previously published anatomical and forensic studies, while recognising its limitations for precise chronological age estimation. Representative examples of the three stages are shown in Figure 1.

#### 2.3.2. Xiphisternal Junction

**Figure 2:**
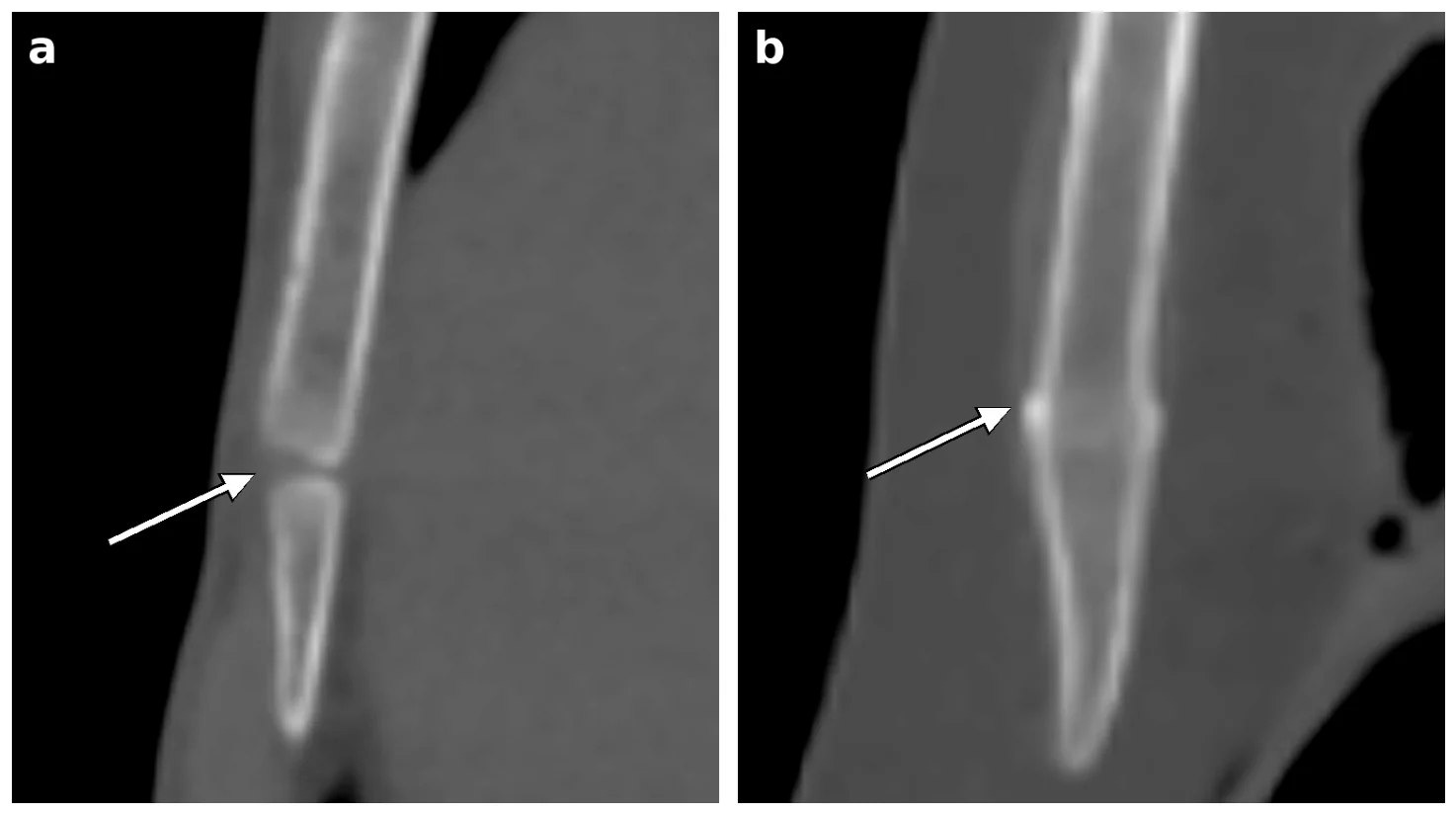
Representative sagittal CT images of the xiphisternal junction. (**a**) Non-fused junction (SK−): absence of osseous continuity between the sternal body and the xiphoid process. (**b**) Fused junction (SK+): complete osseous fusion across the junction. Arrows indicate the xiphisternal junction.

Fusion of the xiphisternal junction was evaluated dichotomously, as non-fused (SK−; absence of osseous continuity between the sternal body and the xiphoid process) or fused (SK+; complete osseous fusion across the junction). When the xiphoid process was entirely cartilaginous and therefore not visible as ossified tissue on CT, the junction was scored as non-fused (SK−). Junctions in which ossification had begun but did not yet extend across the entire junction (partial fusion) met neither definition; these cases were excluded from the study (Section 2.1) rather than being assigned to one of the two categories. Given the morphological characteristics of the xiphisternal joint and its typically binary appearance on CT, further sub-staging was not attempted. Representative examples of a non-fused and a fused xiphisternal junction are shown in Figure 2.

### 2.4. Observer Reliability

All CT examinations were evaluated by one observer (Observer 1), a radiologist, whose scores were used in all analyses. Observer reliability was assessed in a subsample of 25 cases (15 males and 10 females; age range 34–60 years), which included all three manubriosternal stages (12, 7, and 6 cases with Stages 1, 2, and 3, respectively) and both xiphisternal categories (18 fused and 7 non-fused junctions). To assess interobserver reliability, these cases were evaluated independently by a second observer (Observer 2), a forensic medicine specialist, and the evaluations of the two observers were compared. To assess intraobserver reliability, Observer 1 repeated the evaluation of the same cases after a two-week interval, blinded to the initial results. For each comparison, the number of cases with identical scores is reported. Agreement for the dichotomous xiphisternal status was quantified using Cohen’s kappa (κ). For the ordinal manubriosternal stage, a linearly weighted kappa (κ_w_) was used, which gives partial credit to disagreements between adjacent stages [36]. The 95% confidence intervals (CIs) of κ and κ_w_ were obtained by percentile bootstrap resampling of cases (10,000 resamples).

The assessment of observer reliability followed the methodological recommendations of Ferrante and Cameriere for forensic age estimation studies [37], and κ values were interpreted according to the criteria of Landis and Koch [38]: 0.81–1.00, almost perfect agreement; 0.61–0.80, substantial agreement; and 0.41–0.60, moderate agreement. The κ values are reported in the Results section.

### 2.5. Age Stratification Strategy

Chronological age was analysed both as a continuous variable and using five ordered adult age bands: 30–40, 40–50, 50–55, 55–60, and 60–65 years. Age was recorded in completed years, and band limits were lower-inclusive and upper-exclusive (e.g., the 40–50-year band included individuals aged 40 to 49 years), except for the uppermost band, which included individuals aged 60 to 65 years. The band structure was defined a priori, before the fusion data were analysed. Ten-year bands were used below 50 years, the range in which previous studies have described the onset of xiphisternal fusion in the fourth decade and its subsequent increase [9,10,25,27]. From 50 years onward, narrower 5-year bands were adopted for three reasons: (i) 50 years marks the conventional lower boundary of the “old adult” category in anthropological age-at-death reporting [39] and the age beyond which conventional adult skeletal indicators show wide error margins [28]; (ii) previous studies have reported the highest frequencies of sternal fusion, together with persistent non-union, in the sixth and seventh decades [15,18,40], so finer resolution was needed to determine whether xiphisternal fusion prevalence continues to rise or reaches a plateau, and whether non-union persists beyond 60 years; and (iii) the limits of the upper bands coincide with the age thresholds examined in the threshold-based analyses (Section 2.6), so that the descriptive and threshold-based results can be interpreted together. Because narrower bands contain fewer individuals, fusion prevalences are reported with 95% CIs, and a sensitivity analysis with equal-width 10-year bands (30–40, 40–50, 50–60, and 60–65 years, the last band being limited by the upper age bound of the sample) was performed. The non-parametric comparisons, the logistic regression on continuous age, and the threshold-based analyses do not depend on the band structure.

### 2.6. Statistical Analysis

Given the ordinal nature of manubriosternal fusion and the pronounced biological variability of adult skeletal maturation, no assumption of linearity between fusion stage and age was imposed. Differences in age distributions across manubriosternal fusion stages were evaluated using the Kruskal–Wallis test, with effect size quantified using epsilon-squared (ε^2^ = H/[*n* − 1]). For the dichotomous xiphisternal fusion status, age distributions between fused and non-fused groups were compared using the Mann–Whitney *U* test, with effect size expressed as the rank-biserial correlation.

Fusion distributions across the ordered age bands were examined descriptively and compared using chi-square tests of independence. Prevalences of complete manubriosternal fusion and of xiphisternal fusion within each band are reported with Wilson score 95% CIs [41], and these analyses were repeated with equal-width 10-year bands as a sensitivity analysis (Section 2.5). Monotonic trends in manubriosternal fusion were evaluated by logistic regression of complete fusion (Stage 3) on the ordinal age-band score, and xiphisternal fusion status was additionally regressed on continuous age; these analyses were applied without implying deterministic age classification. Associations between sex and fusion were assessed using chi-square tests of independence (with Yates’ continuity correction for 2 × 2 tables) and by logistic regression of fusion status on age and sex.

To assess the forensic relevance of sternal fusion within a probabilistic framework, two complementary analytical approaches were applied. First, to translate fusion status into a forensically applicable format, binary logistic regression models were constructed to assess the association between xiphisternal fusion status and the probability of being at or above three predefined age thresholds (≥50, ≥55, and ≥60 years), and odds ratios (ORs) with 95% CIs were calculated. Diagnostic performance was then assessed with fusion status as a binary test: a fused xiphisternal junction (SK+) was defined as a positive test result and a non-fused junction (SK−) as a negative result, and age at or above the threshold was the target condition. Accordingly, true positives (TP) were individuals with SK+ aged at or above the threshold, false positives (FP) individuals with SK+ below the threshold, false negatives (FN) individuals with SK− at or above the threshold, and true negatives (TN) individuals with SK− below the threshold. Sensitivity, specificity, positive predictive value (PPV), and negative predictive value (NPV) were calculated with Wilson score 95% CIs [41], and positive and negative likelihood ratios (LR+ = sensitivity/[1 − specificity]; LR− = [1 − sensitivity]/specificity) with 95% CIs computed on the logarithmic scale [42]. Because PPV and NPV depend on the proportion of individuals above the threshold in the sample, whereas LRs are not directly affected by this proportion, LRs were regarded as the primary measure of the evidential value of fusion status [43]. To illustrate their practical meaning, post-test probabilities were derived from the LRs for selected pre-test probabilities using Bayes’ theorem (post-test odds = pre-test odds × LR).

The thresholds were selected a priori. The 50-year threshold corresponds to the lower boundary of the old-adult category in anthropological reporting [39] and to the age beyond which conventional adult indicators lose precision [28]. The 55- and 60-year thresholds lie within an age window that has been described as medico-legally relevant in a CT study of sternal fusion [28] and in which the highest frequencies of sternal fusion have been reported [15,18]. In living individuals, the question of whether an adult has reached a given age in the sixth or seventh decade may arise in legal proceedings when reliable documents are lacking, for example in disputes over registered age or age-related entitlements [1,15]; in unidentified deceased persons, such thresholds help to narrow the age range of the biological profile. The thresholds also coincide with the limits of the upper age bands and avoid arbitrary fine-grained classification in mature adulthood.

Second, to evaluate whether combined consideration of both anatomical regions improved forensic discrimination, a logistic regression model incorporating both xiphisternal fusion status and manubriosternal fusion stage was compared against the xiphisternal-only model for the ≥55-year threshold, using the likelihood ratio test, Akaike information criterion (AIC), and area under the receiver operating characteristic curve (AUC). This combined model was specified post hoc and is regarded as exploratory. Its apparent AUC was corrected for optimism by bootstrap resampling (5000 resamples) [44], and 95% CIs for the AUCs were obtained by bootstrap resampling of cases (50,000 resamples); no independent validation sample was available.

All statistical analyses were performed using SPSS software (version 17.0; SPSS Inc., Chicago, IL, USA). Wilson score intervals, likelihood ratios and their CIs, kappa statistics with bootstrap CIs, bootstrap optimism correction, and the equal-width sensitivity analysis were computed in Python (version 3.13) using SciPy (version 1.18), statsmodels (version 0.15), and scikit-learn (version 1.9). A two-sided *p*-value of <0.05 was considered statistically significant. The analytical framework was intentionally designed to avoid deterministic or point-based age estimation; instead, threshold- and probability-based approaches were applied to evaluate how sternal fusion modifies age likelihoods across adulthood.

## 3. Results

### 3.1. Study Population and Age Distribution

The study population comprised 512 individuals (279 males and 233 females) aged between 30 and 65 years. For age-band-based analyses, individuals were stratified into five ordered adult age groups: 30–40 (*n* = 157), 40–50 (*n* = 161), 50–55 (*n* = 54), 55–60 (*n* = 66), and 60–65 years (*n* = 74). The largest proportion of the sample was concentrated in the 30–40- and 40–50-year bands, while progressively narrower distributions were observed in the older age bands, allowing evaluation of gradual age-related shifts rather than binary age separation. Table 1 summarises the distribution of the study population across age bands.

### 3.2. Observer Reliability

In the reliability subsample (*n* = 25), interobserver agreement was complete for the manubriosternal stage (identical scores in 25/25 cases; κ_w_ = 1.00, with the bootstrap CI degenerate at 1.00 because the observers never disagreed) and substantial for the xiphisternal status (23/25 cases; κ = 0.80, 95% CI 0.48–1.00). Intraobserver agreement was almost perfect for both junctions (manubriosternal stage: 24/25 cases, κ_w_ = 0.95, 95% CI 0.83–1.00; xiphisternal status: 24/25 cases, κ = 0.90, 95% CI 0.66–1.00). Owing to the small size of the subsample, the CIs were wide.

### 3.3. Manubriosternal Fusion Patterns Across Age Bands

Manubriosternal fusion stages were observed in all age bands, with substantial overlap across stages. In the youngest age band (30–40 years), most individuals exhibited no fusion (Stage 1; Figure 1a; 72.0%), although partial (13.4%) and complete fusion (14.6%; Figure 1c) were already present in a notable minority. With advancing age, the prevalence of complete manubriosternal fusion (Stage 3) increased from 14.6% (95% CI 10.0–21.0) in the 30–40-year group to 18.5–31.1% in the older bands, reaching 31.1% (95% CI 21.7–42.3) in the 60–65-year group, while the proportion with no fusion decreased from 72.0% to 32.4% over the same range. Despite this overall trend, all three fusion stages were present in every age band. A Kruskal–Wallis test indicated a statistically significant but weak association between manubriosternal fusion stage and chronological age (*H* = 30.51, *p* < 0.001; ε^2^ = 0.060), consistent with the pronounced interindividual variability observed. The detailed distribution of manubriosternal fusion stages by age band is presented in Table 2, and representative CT images of the three fusion stages are shown in Figure 1.

An ordered trend analysis (logistic regression of complete fusion on ordinal age-band score) confirmed a statistically significant, though modest, monotonic trend (β = 0.190, *p* = 0.013), consistent with the extensive overlap observed and indicating that manubriosternal fusion, while formally associated with age, retains limited practical value for individual-level age discrimination.

### 3.4. Xiphisternal Fusion Patterns Across Age Bands

In contrast, xiphisternal fusion demonstrated a clear and progressive age-related pattern. In the 30–40-year band, 62.4% of individuals showed no xiphisternal fusion, whereas 37.6% were fused. With advancing age, the prevalence of xiphisternal fusion increased markedly, reaching 57.8% in the 40–50-year band, 68.5% in the 50–55-year band, and peaking at 77.3% (95% CI 65.8–85.7) in the 55–60-year band. In the oldest band (60–65 years), prevalence plateaued at 75.7% (95% CI 64.8–84.0), indicating that a persistent minority of individuals (approximately one quarter) remained non-fused even beyond 60 years of age. This progressive shift, followed by a plateau, indicates a more orderly but ultimately incomplete age-related behaviour at the xiphisternal junction compared with the manubriosternal junction. The CIs of the three upper bands overlapped widely, reflecting the smaller numbers of individuals in these bands. The distribution of xiphisternal fusion status by age band is detailed in Table 3.

A Mann–Whitney *U* test confirmed a highly significant difference in age between fused and non-fused individuals (*U* = 20,098, *p* < 0.001; rank-biserial correlation = 0.371), and a logistic regression of fusion status on continuous age showed a significant positive association (OR = 1.07 per year, 95% CI 1.05–1.09, *p* < 0.001).

In the sensitivity analysis with equal-width 10-year bands (Table S3), the same pattern was observed. Xiphisternal fusion prevalence increased from 37.6% (30–40 years) to 57.8% (40–50 years) and 73.3% (95% CI 64.8–80.4; 50–60 years) and was 75.7% at 60–65 years (χ^2^ = 47.89, df = 3, *p* < 0.001), whereas complete manubriosternal fusion remained between 14.6% and 20.5% below 60 years and reached 31.1% at 60–65 years (ordered trend β = 0.269, *p* = 0.011).

### 3.5. Threshold-Based Analysis of Xiphisternal Fusion

To translate these findings into a forensically applicable format, logistic regression models were constructed to assess the association between xiphisternal fusion status and the probability of exceeding three predefined adult age thresholds (≥50, ≥55, and ≥60 years). Presence of xiphisternal fusion was associated with significantly increased odds of exceeding each threshold: OR = 3.15 (95% CI 2.13–4.64, *p* < 0.001) for ≥50 years; OR = 3.14 (95% CI 2.02–4.87, *p* < 0.001) for ≥55 years; and OR = 2.57 (95% CI 1.46–4.51, *p* = 0.001) for ≥60 years. Diagnostic performance at each threshold is summarised in Table 4. Sensitivity was similar across thresholds (74.2–76.4%), whereas specificity was low (45.2–52.2%). PPV decreased from 48.6% at ≥50 years to 18.9% at ≥60 years, and NPV increased from 76.9% to 91.7%, reflecting the decreasing proportion of individuals above the higher thresholds in this sample (37.9%, 27.3%, and 14.5%, respectively) rather than a change in the behaviour of the marker. LR+ ranged from 1.38 (95% CI 1.18–1.61) at ≥60 years to 1.55 (95% CI 1.35–1.79) at ≥50 years, and LR− from 0.48 (95% CI 0.35–0.66) at ≥55 years to 0.54 (95% CI 0.36–0.81) at ≥60 years. Observing a fused junction therefore multiplied the odds of being at or above a threshold by approximately 1.4–1.6, whereas observing a non-fused junction approximately halved them. For the ≥55-year threshold, for example, a pre-test probability of 50% increased to 60.1% after observing a fused junction and decreased to 32.4% after observing a non-fused junction. These results confirm that xiphisternal fusion shifts the probability of belonging to an older adult age category, but only modestly, because fused and unfused junctions co-existed within every age band; fusion status alone cannot classify an individual as being above or below a given threshold. It should instead be interpreted as a probabilistic modifier of age likelihood rather than a deterministic classifier.

### 3.6. Comparative Age-Related Behaviour of the Two Junctions

**Figure 3:**
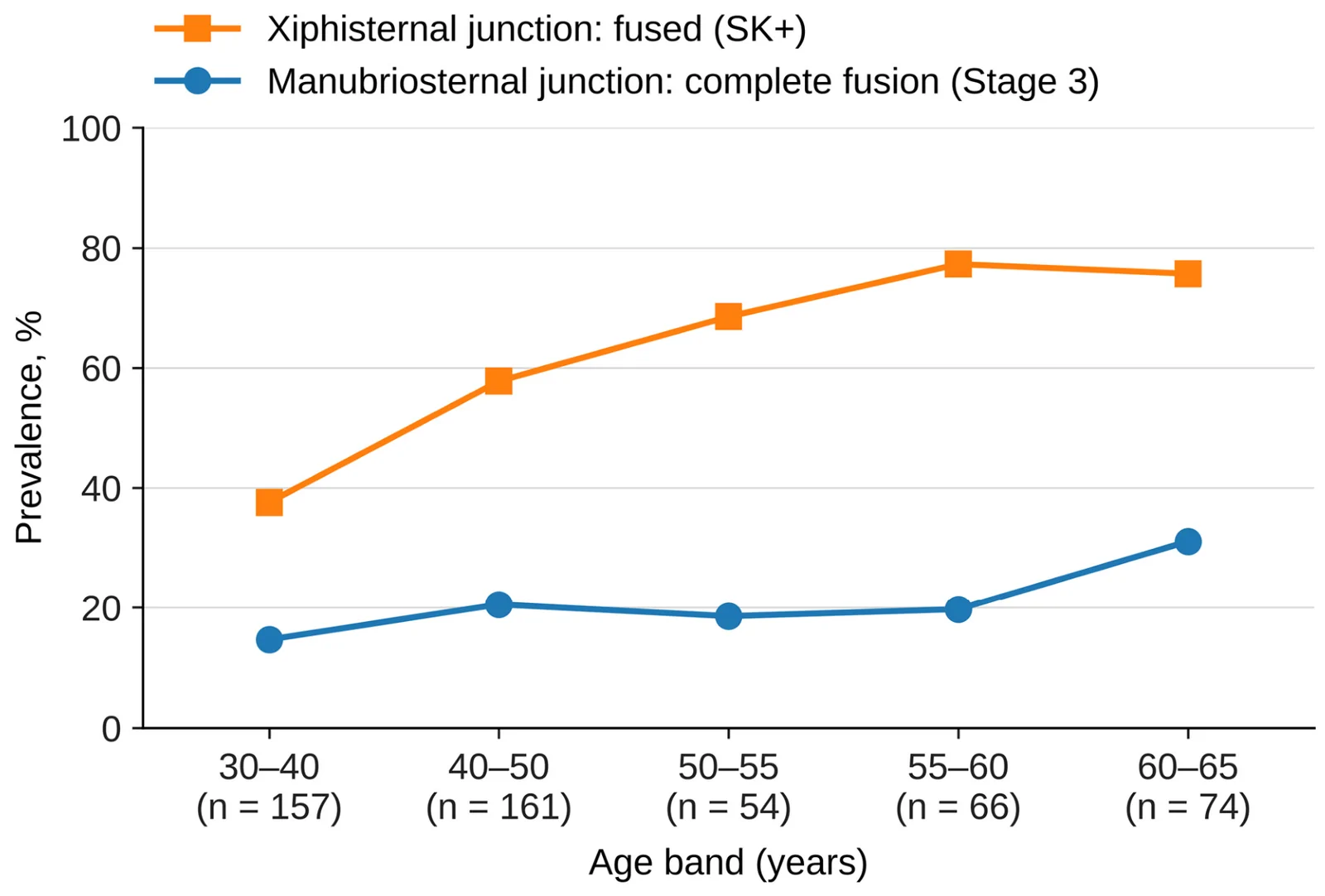
Age-related changes in sternal fusion patterns: prevalence of xiphisternal fusion (SK+, orange squares) and of complete manubriosternal fusion (Stage 3, blue circles) across age bands.

Direct comparison of the two junctions revealed distinct age-related behaviours. While both manubriosternal and xiphisternal fusion frequencies increased with age, the magnitude and clarity of this increase differed substantially. Manubriosternal fusion showed only a modest and irregular rise with persistent overlap between stages, whereas xiphisternal fusion displayed a steep increase followed by a plateau after age 55. This contrast is illustrated in Figure 3, which depicts the prevalence of complete manubriosternal fusion (Stage 3) and xiphisternal fusion (SK+) across the five age bands. When both junctions were combined in a single logistic regression model predicting the ≥55-year threshold, discrimination improved significantly relative to the xiphisternal-only model (likelihood ratio test, χ^2^ = 12.57, df = 2, *p* = 0.002; AUC 0.628 [95% CI 0.584–0.671] to 0.676 [95% CI 0.629–0.727]; AIC 576.0 to 567.4). After bootstrap correction for optimism, the AUC of the combined model was 0.669. Although statistically significant, this improvement was small; an AUC below 0.70 indicates limited discrimination, and the exploratory model was not validated in an independent sample.

### 3.7. Exploratory Analysis of Sex Differences

Exploratory sex-stratified analyses were performed for both the manubriosternal and xiphisternal junctions, and the results are presented in Tables S1 and S2 (Supplementary Materials). Xiphisternal fusion prevalence was broadly similar between males and females across most age bands (e.g., 30–40 years: 38.4% in males vs. 36.6% in females; 55–60 years: 81.1% vs. 72.4%), and a logistic regression of fusion status on age and sex showed no statistically significant independent effect of sex (OR for males vs. females = 1.32, 95% CI 0.91–1.92, *p* = 0.14). Similarly, chi-square tests of independence found no significant association between sex and xiphisternal fusion status (χ^2^ = 1.68, *p* = 0.20) or manubriosternal fusion stage (χ^2^ = 2.83, *p* = 0.24) overall. In accordance with the predefined study design, and because the primary objective of the study was to evaluate the overall probabilistic behaviour of sternal fusion across adulthood, the principal analyses were performed using the combined study population.

### 3.8. Comparative Synthesis of Junction Behaviour

Taken together, these results demonstrate distinct age-related behaviours at the two sternal junctions. Xiphisternal fusion shows a consistent but modest probability shift toward older adult age thresholds (LR+ 1.38–1.55; LR− 0.48–0.54), plateauing at approximately 75–77% beyond age 55, whereas manubriosternal fusion stages remain broadly overlapping and only weakly informative despite reaching statistical significance. Combined consideration of both junctions slightly improved discrimination in an exploratory model. This contrast underscores the importance of treating sternal fusion as a probabilistic modifier rather than a deterministic age estimator, and suggests that multi-region assessment may be preferable to single-junction evaluation.

## 4. Discussion

### 4.1. Principal Findings

The present findings reaffirm a central challenge in adult skeletal age assessment: biological variability is not noise to be eliminated, but an intrinsic feature of post-growth maturation. Both manubriosternal and xiphisternal junctions demonstrated wide overlap across adult age ranges, reflecting the complex interplay of mechanical loading, hormonal influences, metabolic factors, genetic background, and individual life history reported in previous anatomical, radiographic, and CT-based studies [5,6,7,8,9,10,11,12,13,20,21,22,23,24,25,26].

Although the associations between fusion and age were statistically significant, their magnitude was modest. The LRs associated with xiphisternal fusion status (approximately 1.4–1.6 for a fused and approximately 0.5 for a non-fused junction) and the AUC of the exploratory combined model (0.676) indicate limited discrimination. In a sample of this size, statistical significance should therefore not be equated with practical forensic applicability. The main contribution of the present study lies less in identifying a new age indicator than in quantifying, in a large CT sample, the actual discriminatory performance of an established one and thereby defining its limits.

The heterogeneous findings reported in the literature most likely reflect several interacting factors rather than a single source of variation [31,32]. Interindividual biological variability, inter-population differences, study design, sample composition, imaging modality, and staging criteria may all contribute to the observed inconsistencies. Attempts to interpret sternal fusion within deterministic or quasi-linear age-estimation frameworks have therefore produced heterogeneous forensic conclusions [5,6,7,8,9,10,11,12,13,20,21,22,23,24,25,26]. These differences should not be interpreted as being caused solely by the analytical approach. Rather, biological variability determines the observed overlap, while the analytical framework influences how such overlap is interpreted in forensic practice. When fusion stages are implicitly treated as temporal milestones, overlap is perceived as methodological failure. Conversely, when uncertainty is explicitly acknowledged, the same overlap may still provide limited supplementary information on age without supporting deterministic age estimation.

### 4.2. Comparison with Previous Studies

Manubriosternal fusion showed only a modest and irregular increase with advancing age; although this association reached statistical significance given the sample size, the effect size was small and all three fusion stages remained present across every age band. This pattern is consistent with previous anatomical, radiographic and CT-based studies reporting early, late, or absent fusion in adults of comparable age ranges [5,6,7,8,9,12,13,22,25]. In the present sample, complete fusion was already present in 14.6% of individuals aged 30–40 years but in only 31.1% of those aged 60–65 years. Comparable findings have been obtained with different methods: in a CT study of 734 Indian patients, complete manubriosternal fusion did not exceed 44% in any decade and its discrimination was moderate (AUC 0.642) [28]; in a Turkish CT sample aged 30–80 years, the incidence of complete fusion did not differ between age groups [21]; and in Japanese postmortem CT and Croatian multi-slice CT (MSCT) data, manubriosternal fusion showed no significant association with age [20,45]. In autopsy and skeletal samples, the junction remained unfused in many elderly individuals [8,9,10,25], whereas complete fusion was observed in individuals as young as 25 years [8]; in a small North Indian autopsy series that also included adolescents, manubriosternal fusion was observed predominantly after 35 years of age [19]. A likely explanation lies in the heterogeneous biological mechanisms underlying manubriosternal synostosis. Prior work has distinguished between matrical primary fusion, which may occur independently of age, and sclerotic secondary fusion, which is more plausibly age-related [7,8,9]. Current imaging modalities, including thin-slice CT, cannot reliably differentiate between these processes [20]. Consequently, manubriosternal fusion provides little information on age when considered in isolation. Even complete fusion cannot be interpreted as indicative of a specific adult age range, nor does absence of fusion reliably exclude advanced adulthood [5,6,7,8,9,25]. These findings support the growing consensus that manubriosternal fusion should not be used as a standalone criterion for adult age estimation in forensic practice [7,8,9,25].

In contrast, xiphisternal fusion exhibited a clear and statistically significant increase in prevalence across ordered adult age bands up to the 55–60-year band, followed by a plateau. This behaviour aligns with several CT-based studies reporting higher fusion rates with advancing age and suggests a more orderly age-related pattern at the xiphisternal junction compared with the manubriosternal junction [20,21,22,23,24,26]. With respect to timing, previous studies place the onset of xiphisternal fusion in the fourth decade: no fusion was observed below 30 years in South Indian and Thai autopsy samples [9,10] or below 25 years in a North Indian autopsy series [19], and fusion was reported to start between 30 and 39 years in a Mediterranean skeletal sample [25]. In a living Indian CT population aged 35–50 years, the mean age of individuals with complete fusion was approximately 46 years, compared with approximately 38 years in those with unfused junctions [27]. Later mean ages were reported in other Indian samples: on lateral sternal radiographs, the mean age of individuals with xiphisternal fusion was 54.3 years in males and 57.9 years in females [15], in a large Central Indian autopsy series, fusion began at mean ages of 43.7 and 49.7 years in males and females, respectively, and 24 of 52 individuals older than 60 years showed no fusion [18]; and in Thai autopsy material, unfused xiphisternal junctions were observed up to 76 years in females and 78 years in males [10]. The 37.6% prevalence of xiphisternal fusion in our 30–40-year band is consistent with an onset in the fourth decade, and the persistence of non-union in approximately one quarter of individuals aged 60–65 years agrees with the latter observations but contrasts with a South Indian autopsy series in which non-fusion was not observed after 48 years in males and 46 years in females [9]. These differences probably reflect population, sample size, and methodological factors, including direct inspection versus imaging and the handling of partial fusion, which was counted as fusion in the Thai series [10] but excluded in the present study. Recent studies, including CT-based work in an Egyptian population and a skeletal study of a Mediterranean population, similarly demonstrated age-related increases in sternal fusion while emphasising the persistence of substantial overlap between chronological age groups [25,26]. Importantly, however, this apparent regularity does not translate into age specificity. Fused and unfused xiphisternal junctions co-existed across all age bands, including both younger and older adults [20,21,22,23,24,26]. This observation reconciles seemingly contradictory conclusions in the literature, where statistically significant correlations have been interpreted either as evidence of forensic utility or dismissed due to overlap. Our findings demonstrate that both interpretations can be correct, depending on the analytical framework applied. The plateau in xiphisternal fusion prevalence observed beyond 55 years in the present cohort is consistent with a recent Egyptian multidetector CT (MDCT)-based study, which reported that full sternal fusion was reached by approximately 67 years in 97% of females, with males reaching this stage roughly a decade later, underscoring that even comprehensive multi-element sternal scoring systems require a substantially older cohort before near-complete fusion is observed [40]. Where discriminatory performance was reported, it was similarly limited when sternal fusion or related chest-plate indicators were used alone or in simple composites, with standard errors of estimate of 12–15 years [20], mean absolute errors of 7–10 years [24,30], and 95% prediction intervals approximately 42 years wide [45]. Higher discrimination (AUC 0.896 for ≥45 years) was reported for combined xiphisternal and manubriosternal fusion in an autopsy series that included individuals from 15 years of age [19]; the inclusion of adolescents and young adults, in whom unfused junctions predominate, may inflate discrimination relative to a 30–65-year sample such as ours. Table 5 summarises the principal previous studies together with the present results.

### 4.3. Practical Implications for Forensic Age Assessment

When viewed probabilistically, xiphisternal fusion does not define an age threshold but shifts the likelihood distribution modestly toward older adulthood. The presence of fusion increases the probability of exceeding higher adult age thresholds, whereas its absence shifts probability toward younger adulthood without excluding older ages [3,4]. The size of this shift is small: for the ≥55-year threshold, a fused junction raises a pre-test probability of 50% to approximately 60% and a non-fused junction lowers it to approximately 32%; even with a pre-test probability of 75%, the post-test probability after observing fusion is only approximately 82% (Table 4). Population-level associations therefore translate into individual probability estimates that remain broad, and sternal fusion cannot by itself support the conclusion that an individual is above or below a given age.

From a practical forensic perspective, these findings indicate that sternal fusion should not be interpreted as providing a definitive estimate of chronological age. Rather, xiphisternal fusion may contribute supportive age-related information when evaluated together with other validated skeletal indicators and case-specific findings. Likewise, the absence of fusion should not be interpreted as excluding older adulthood. Such an interpretation better reflects the biological variability observed in adult skeletal maturation and supports transparent communication of uncertainty in forensic reporting.

Realistic scenarios in which sternal fusion may provide supplementary information include: (i) the examination of unidentified deceased persons for whom postmortem CT is available, where the sternum is included in routine thoracic scans and can be scored at no additional cost alongside indicators such as the pubic symphysis, the sternal rib ends, and costal cartilage ossification [2,3,4,20]; (ii) living individuals whose age is disputed and in whom thoracic CT has already been performed for a clinical indication, for example after trauma; and (iii) multifactorial assessments in which a prior age distribution derived from other indicators is updated with the LRs reported here. In each scenario, sternal fusion should be considered a complementary indicator rather than an independent method of age estimation, and its weight should be small relative to more informative indicators. Because indicators of the thoracic cage may reflect shared biological processes, their LRs should not simply be multiplied as if they were conditionally independent; joint modelling, as in the combined model explored here, is preferable [45].

A recurring limitation in the sternal fusion literature is the implicit transfer of anthropological staging concepts into forensic decision-making without adequate consideration of uncertainty [1,2]. Classical skeletal methods were developed to describe population-level trends, yet forensic practice requires individual-level interpretation with transparent communication of uncertainty. From this perspective, the relevant forensic question is not “What is the individual’s age?” but rather “How does this observation modify the likelihood of competing age hypotheses?” In the present study, xiphisternal fusion modestly alters the probability of belonging to older adult age categories, whereas manubriosternal fusion contributes little age-related information. This distinction is critical for responsible forensic reporting [3,4].

Although the present analysis did not implement full Bayesian age estimation, the LRs in Table 4 allow the findings to be used within a Bayesian framework: observation of xiphisternal fusion increases the posterior probability of older age thresholds relative to the prior, while absence of fusion has the opposite effect [3,4]. Importantly, the magnitude of this probability shift depends on the prior context and should be integrated with other skeletal indicators, imaging findings, and case-specific information rather than interpreted in isolation.

These considerations also bear on radiation protection. The present data were obtained retrospectively from CT examinations acquired for clinical indications, so their evaluation involved no additional radiation exposure. The prospective acquisition of thoracic CT solely for age assessment is a different matter: radiological examinations without a medical indication require a specific legal basis and must satisfy the principles of justification and optimisation [1,46]. Reported doses for radiographic procedures used in forensic age estimation range from less than 0.1 µSv for a hand radiograph to more than 800 µSv for CT [47], and diagnostic CT examinations typically entail effective doses of approximately 2–20 mSv [48]. Given the modest discriminatory performance observed here, the expected information gain would not justify thoracic CT acquired exclusively to assess sternal fusion; the approach should be limited to CT data already available for clinical or postmortem purposes.

### 4.4. Strengths and Limitations

A major strength of this study lies in its CT-based evaluation of both sternal junctions within a unified analytical framework that explicitly rejects deterministic age claims. Observer agreement in the reliability subsample was substantial to almost perfect, suggesting that the observed variability largely reflects biological variation rather than measurement error; however, agreement concerns the reproducibility of the scoring rather than the validity of the predictive models, and it was assessed in a small subsample. The use of ordered age bands and threshold-based analyses avoids artificial dichotomisation and aligns more closely with forensic reasoning [3,4].

Several limitations warrant consideration. The retrospective design and single-population sample may limit generalisability, and sex-specific effects were explored only descriptively [7,8]. Although exploratory sex-stratified analyses did not demonstrate consistent differences between males and females within the present cohort, the study was not specifically designed or powered to establish sex-specific reference standards. Larger multicentre studies with balanced sex distributions are required to determine whether subtle sex-related differences exist in sternal fusion patterns across different populations. Notably, a recent Egyptian MDCT study of 247 individuals found that males reached full sternal fusion approximately a decade later than females despite stronger age-score correlations in males [40], suggesting that sex-related differences in sternal maturation may only become apparent in cohorts extending well beyond the 65-year upper limit of the present study.

Cases with partial xiphisternal fusion were excluded to allow a dichotomous classification. Because such transitional cases may cluster at intermediate ages, their exclusion may have accentuated the contrast between fused and non-fused groups (spectrum bias); the reported prevalences and diagnostic measures therefore apply to individuals with unambiguous fusion status, and performance in routine casework, in which partial fusion is encountered, may be lower. In addition, scoring an entirely cartilaginous xiphoid as non-fused reflects the absence of osseous continuity but does not distinguish delayed ossification from persistent non-union.

Observer reliability was assessed in a subsample of only 25 cases, which limits the precision of the agreement estimates; for interobserver agreement on xiphisternal status (κ = 0.80), the lower 95% confidence limit (0.48) is compatible with only moderate agreement. Moreover, because cases with partial xiphisternal fusion had been excluded, agreement could not be assessed for these transitional cases, in which classification is likely to be most difficult, so the reproducibility of the scoring in routine casework may be lower than observed here.

The upper 5-year bands contained fewer individuals (54–74) than the lower 10-year bands (157–161), so the prevalence estimates in these bands are less precise, as reflected in their wider CIs; the sensitivity analysis with equal-width bands nevertheless yielded the same pattern, and the threshold-based analyses do not depend on the band structure. PPV and NPV depend on the age composition of the present sample and should not be transferred directly to other settings; LRs are more transferable but may still vary with the age distribution of the reference sample.

The age range was extended to 65 years to examine whether non-union persists into the seventh decade. Xiphisternal fusion prevalence reached a plateau at approximately 75–77% from age 55 onward, indicating that a persistent minority of individuals (approximately one quarter) remain non-fused even beyond 60 years. Future studies including even broader age ranges are required to determine whether this plateau persists into more advanced adulthood and how this affects forensic interpretation.

Another limitation relates to the qualitative categorisation of partial manubriosternal fusion. Although a three-stage classification was selected to maintain comparability with previous studies, partial fusion encompasses a wide spectrum of osseous bridging that may not be adequately captured by a single intermediate category. Future CT-based investigations should explore quantitative approaches, such as measuring the proportion of the fused articular surface or the absolute extent of osseous bridging relative to the total joint interface. Such continuous imaging variables may better characterise the gradual nature of sternal fusion and improve probabilistic modelling of adult skeletal ageing.

Additionally, other components of the sternal plate, such as costal cartilage ossification, were not assessed and may contribute additional information in composite models [11,20,35]. Future research should prioritise integrative, multimodal approaches that combine sternal fusion with quantitative imaging features and formal probabilistic inference.

The combined model was specified post hoc and was not validated in an independent sample. Interobserver agreement and model validation address different aspects of reliability: agreement between observers supports the reproducibility of the observations but does not protect a model against optimism, that is, the overestimation of its discriminatory performance when it is evaluated on the data used to develop it [44]. The bootstrap-corrected AUC (0.669) was only slightly lower than the apparent AUC (0.676), but the model should be regarded as exploratory until it has been validated externally.

### 4.5. Future Directions

Although the two junctions were characterised separately as the primary analytical strategy, a post hoc combined logistic regression incorporating both xiphisternal fusion status and manubriosternal fusion stage significantly outperformed the xiphisternal-only model for predicting the ≥55-year threshold (likelihood ratio test, *p* = 0.002; AUC improved from 0.628 to 0.676). This finding suggests that joint, multi-region assessment may modestly improve the discriminatory value of sternal fusion, although the resulting AUC remains below 0.70. This is consistent with a recent Croatian MSCT-based study that combined manubrio-mesosternal and xiphisternal fusion scoring with costal cartilage ossification staging into multivariable prediction models with formally validated prediction intervals, reporting improved age-estimation performance over single-marker approaches [45]. Larger prospective studies should formally validate combined multi-region models of this kind, ideally incorporating additional skeletal indicators such as costal cartilage ossification alongside sternal fusion.

The present findings underscore the need to recalibrate expectations regarding adult skeletal age assessment. Rather than seeking incremental gains from isolated morphological markers, future progress is more likely to arise from frameworks that explicitly model uncertainty and integrate multiple sources of evidence [1,3,4]. Within such frameworks, sternal fusion—particularly at the xiphisternal junction—can serve as a minor contributor to age assessment, provided that its limited precision is acknowledged [11,20].

## 5. Conclusions

This study demonstrates that sternal fusion, when assessed by CT, should not be used as a standalone method for deterministic adult age estimation in forensic practice. Manubriosternal fusion exhibits substantial interindividual variability with persistent overlap across adult age ranges, providing little age-related information when considered in isolation. In contrast, xiphisternal fusion demonstrates a clear age-related increase in prevalence; however, the considerable overlap between fused and non-fused individuals precludes precise chronological age estimation, and its likelihood ratios (approximately 1.4–1.6 for fusion and 0.5 for non-fusion) indicate only modest shifts in age probability.

Accordingly, neither manubriosternal nor xiphisternal fusion should be interpreted as providing a definitive estimate of chronological age. Rather, xiphisternal fusion may contribute modest supplementary information when interpreted probabilistically and integrated with other validated skeletal indicators, imaging findings, and case-specific evidence within a comprehensive forensic assessment, using CT images that are already available for clinical or postmortem purposes rather than images acquired for this purpose.

By adopting a probabilistic and threshold-based framework, the present study quantifies the limited evidential value of sternal fusion while avoiding unwarranted deterministic conclusions. These findings emphasise the importance of transparent communication of uncertainty and support the use of multimodal approaches rather than isolated skeletal markers in adult forensic age assessment. Future studies should include broader age ranges, more diverse populations, quantitative CT-based assessment of fusion patterns, and external validation of combined models to further define the evidential value of sternal fusion within probabilistic forensic age estimation frameworks.

## Figures and Tables

**Table 1:** Distribution of the study population across age bands (*N* = 512).

Group	30–40 Years	40–50 Years	50–55 Years	55–60 Years	60–65 Years	Total
All individuals, *n*	157	161	54	66	74	512
Males, *n*	86	90	24	37	42	279
Females, *n*	71	71	30	29	32	233

Sex-specific counts per age band are derived from Tables S1 and S2 (Supplementary Materials).

**Table 2:** Distribution of manubriosternal fusion stages by age band, *n* (%).

Stage	30–40 Years (*n* = 157)	40–50 Years (*n* = 161)	50–55 Years (*n* = 54)	55–60 Years (*n* = 66)	60–65 Years (*n* = 74)
1 (no fusion)	113 (72.0)	94 (58.4)	32 (59.3)	34 (51.5)	24 (32.4)
2 (partial fusion)	21 (13.4)	34 (21.1)	12 (22.2)	19 (28.8)	27 (36.5)
3 (complete fusion)	23 (14.6)	33 (20.5)	10 (18.5)	13 (19.7)	23 (31.1)
**Stage 3, % (95% CI)**	**14.6** **(10.0–21.0)**	**20.5** **(15.0–27.4)**	**18.5** **(10.4–30.8)**	**19.7** **(11.9–30.8)**	**31.1** **(21.7–42.3)**

Kruskal–Wallis test: *H* = 30.51, *p* < 0.001, ε^2^ = 0.060. Ordered trend (logistic regression on ordinal age-band score): β = 0.190, *p* = 0.013; 95% CIs are Wilson score intervals.

**Table 3:** Distribution of xiphisternal fusion status by age band, *n* (%).

Status	30–40 Years (*n* = 157)	40–50 Years (*n* = 161)	50–55 Years (*n* = 54)	55–60 Years (*n* = 66)	60–65 Years (*n* = 74)
Non-fused (SK−)	98 (62.4)	68 (42.2)	17 (31.5)	15 (22.7)	18 (24.3)
Fused (SK+)	59 (37.6)	93 (57.8)	37 (68.5)	51 (77.3)	56 (75.7)
**SK+, % (95% CI)**	**37.6** **(30.4–45.4)**	**57.8** **(50.0–65.1)**	**68.5** **(55.3–79.3)**	**77.3** **(65.8–85.7)**	**75.7** **(64.8–84.0)**

Mann–Whitney *U* test (age by fusion status): *U* = 20,098, *p* < 0.001, rank-biserial correlation = 0.371. Chi-square test of independence (fusion status × age band): χ^2^ = 48.82, df = 4, *p* < 0.001. 95% CIs are Wilson score intervals.

**Table 4:** Diagnostic performance of xiphisternal fusion (SK+ as positive test) for age at or above the forensically relevant thresholds (*N* = 512).

Measure	≥50 Years	≥55 Years	≥60 Years
Individuals at/above threshold, *n* (%)	194 (37.9)	140 (27.3)	74 (14.5)
TP/FP	144/152	107/189	56/240
FN/TN	50/166	33/183	18/198
Sensitivity, % (95% CI)	74.2 (67.6–79.9)	76.4 (68.8–82.7)	75.7 (64.8–84.0)
Specificity, % (95% CI)	52.2 (46.7–57.6)	49.2 (44.1–54.3)	45.2 (40.6–49.9)
PPV, % (95% CI)	48.6 (43.0–54.3)	36.1 (30.9–41.8)	18.9 (14.9–23.8)
NPV, % (95% CI)	76.9 (70.8–82.0)	84.7 (79.3–88.9)	91.7 (87.2–94.7)
LR+ (95% CI)	1.55 (1.35–1.79)	1.50 (1.31–1.72)	1.38 (1.18–1.61)
LR− (95% CI)	0.49 (0.38–0.64)	0.48 (0.35–0.66)	0.54 (0.36–0.81)
OR (95% CI)	3.15 (2.13–4.64)	3.14 (2.02–4.87)	2.57 (1.46–4.51)

TP, true positives (SK+, age at or above the threshold); FP, false positives (SK+, age below the threshold); FN, false negatives (SK−, age at or above the threshold); TN, true negatives (SK−, age below the threshold); PPV, positive predictive value; NPV, negative predictive value; LR+, positive likelihood ratio; LR−, negative likelihood ratio; OR, odds ratio. Proportions are given with Wilson score 95% CIs and likelihood ratios with 95% CIs computed on the logarithmic scale. PPV and NPV depend on the proportion of individuals at or above each threshold in this sample and are not directly transferable to other settings.

**Table 5:** Summary of previous studies of sternal fusion in relation to adult age, compared with the present study.

Study	Population; *n*; Age (Years)	Material/Method	Junctions; Scoring	Main Age-Related Findings
Chandrakanth et al., 2012 [9]	South India; 118 (67 M, 51 F); 20–80	Autopsy, direct inspection	MS, XS; fused/unfused	Fusion at both junctions increased with age; no XS fusion <30 years; XS non-fusion up to 48 (M)/46 (F) years; MS unfused even in the elderly
Singh and Pathak, 2013 [7]	Northwest India; 343; NR	Autopsy, direct inspection	Fusion of sternal elements among eight non-metric traits	Cases with fused elements increased with age, but the fusion pattern was variable; logistic regression on all eight traits classified 70.0% into the correct age group
Chopra et al., 2014 [15]	North India (living); 200; ≥25	Lateral sternal radiographs	MS, XS; fused/unfused (equivocal excluded)	Earliest fusion at 26 years (XS) and 29 (M)/35 (F) years (MS); mean age with XS fusion 54.3 (M)/57.9 (F) years
Bacci et al., 2018 [8]	Black South African; 461 sterna; adults	Sterna, direct inspection	MS, XS; fusion phases	Most sterna unfused throughout adult life; complete fusion as early as 25 years; logistic regression accuracy 62.5%
Monum et al., 2020 [20]	Japan; 320 (+50 test); adults	3D postmortem CT	MS and XS fusion among five chest-plate scores	MS fusion not significantly correlated with age; best composite score (incl. XS fusion) SEE 12.4 (M)/14.7 (F) years
Bolatlı et al., 2020 [22]	Turkey; 700; NR	MDCT, 3D volume rendering	Sternal morphometry; MS ossification; xiphoid types	MS ossification differed significantly by age and sex
Oktay and Aytaç, 2022 [21]	Turkey; 200; 30–80	Chest CT	MS fusion and degeneration; second costal cartilage	Incidence of complete MS fusion did not differ between age groups; MS degeneration increased with age but with wide ranges
Partido Navadijo and Alemán Aguilera, 2022 [25]	Spain; 189; 20–98	Skeletal collection, direct inspection	MS, XS; three stages	Only XS fusion related predictably to age (onset 30–39 years); MS usually unfused
Kuatrakul and Vachirawongsakorn, 2023 [10]	Thailand; 184 (90 M, 94 F); 20–84	Autopsy, macerated sternum, direct inspection	MS, XS; fused (incl. partial)/unfused	XS fused in 36.4%; youngest fused XS 30 (F)/32 (M) years; unfused XS up to 76 (F)/78 (M) years; fusion of limited value for age
Badhe et al., 2023 [27]	India (living); 384; 35–50	HRCT of the chest	XS; complete fusion vs. unfused	Mean age with complete fusion 46.3 (M)/45.6 (F) years vs. 38.4 (M)/37.9 (F) years when unfused
Tang et al., 2024 [24]	China; 2700; 20–70	CT, maximum intensity projection	Four sternal indicators; data mining	MAE 9.1 (M)/10.4 (F) years (internal validation); 7.1 (M)/7.2 (F) years (external validation)
Vickov et al., 2025 [45]	Croatia; 144 (+36 test); NR	MSCT	MS, XS, costal cartilage ossification	All parameters except MS fusion associated with age; combined model 95% PI width ≈ 42 years
Verma et al., 2025 [17]	North India; 195 (165 M, 30 F); ≥14	Autopsy, radiographs of excised sterna	MS, XS; fused/unfused	Fusion in 23.6% (MS) and 34.4% (XS); earliest at 19 (M)/40 (F) years; moderate, sex-specific associations with age
Nema et al., 2025 [18]	Central India; 770 (432 M, 338 F); ≥10	Autopsy, direct inspection	MS, XS (complete/partial/absent); sternal morphometry	XS fusion began at mean ages of 43.7 (M)/49.7 (F) years; 24/52 individuals >60 years unfused; MS fusion variable
Gehlot and Gehlot, 2026 [28]	India; 734; 0–97	Chest CT	MS; complete fusion vs. no fusion	Complete fusion in 26.4%, ≤44% in any decade; AUC 0.642 (cut-off ≥54 years)
Partido Navadijo et al., 2026 [29]	Spain (Mediterranean); 240; adults	Thoracic CT, 3D images	Costal cartilage ossification (ribs 1–7); sternal synostosis	Regression formulae with high coefficients of determination; synostosis-specific results NR
Sharif et al., 2026 [40]	Egypt; 247; NR	MDCT	MS, XS, sternebrae; three-element staging	~97% of females fully fused by ~67 years, males ~10 years later; performance declined above 40 years
Pradeep and Chawla, 2026 [19]	North India; 115; ≥15	Autopsy, direct inspection	Sternal body, MS, XS	XS fusion associated with age (absent <25 years); MS fusion mainly after 35 years; combined model AUC 0.896 for ≥45 years
Tareeva et al., 2026 [30]	Russia; 175; 18–79	Chest CT, 3D	MS, XS, costosternal joints; scoring scale	Regression model MAE 7.3 years
Present study	Turkey; 512 (279 M, 233 F); 30–65	Clinical thoracic MDCT	MS, three stages; XS, fused/unfused	MS weakly related to age (ε^2^ = 0.060); XS fusion 37.6% (30–40 years) to 75.7–77.3% (≥55 years); LR+ 1.38–1.55, LR− 0.48–0.54; combined model AUC 0.676 (≥55 years)

F, females; M, males; MS, manubriosternal junction; XS, xiphisternal junction; NR, not reported in the abstract (full text not accessible); HRCT, high-resolution CT; MDCT, multidetector CT; MSCT, multislice CT; SEE, standard error of estimate; MAE, mean absolute error; PI, prediction interval; AUC, area under the receiver operating characteristic curve; LR+/LR−, positive/negative likelihood ratio.

## Data Availability

The datasets used and/or analysed during the current study are available from the corresponding author on reasonable request.

## Supplementary Materials

The following supporting information can be downloaded at: https://www.mdpi.com/article/10.3390/diagnostics16213629/s1, Table S1: Manubriosternal fusion stage by age band and sex; Table S2: Xiphisternal fusion status by age band and sex; Table S3: Sensitivity analysis with equal-width 10-year age bands.

## Abbreviations

The following abbreviations are used in this manuscript:

AIC	Akaike information criterion
AUC	Area under the receiver operating characteristic curve
CI	Confidence interval
CT	Computed tomography
FN	False negative
FP	False positive
LR+/LR−	Positive/negative likelihood ratio
MDCT	Multidetector computed tomography
MPR	Multiplanar reconstruction
MSCT	Multislice computed tomography
NPV	Negative predictive value
OR	Odds ratio
PPV	Positive predictive value
SK−/SK+	Non-fused/fused xiphisternal junction
TN	True negative
TP	True positive
