# Supplementary material for: Fusion of the Manubriosternal and Xiphisternal Junctions on Computed Tomography: A Probabilistic and Threshold-Based Approach to Age Assessment
Source: Diagnostics (Basel). 2026 Nov 3;16(21):3629. doi: 10.3390/diagnostics16213629 (PMC13761722; doi:10.3390/diagnostics16213629)
Supplement: Supplementary file 1 [file diagnostics-16-03629-s001.zip › diagnostics-4646844-supplementary.pdf]

Supplementary Materials

Fusion of the Manubriosternal and Xiphisternal Junctions on Computed Tomography: A Probabilistic and Threshold-Based Approach to Age Assessment

Table S1. Manubriosternal fusion stage by age band and sex, *n*.

**Sex/Stage**	**30–40 years**	**40–50 years**	**50–55 years**	**55–60 years**	**60–65 years**
Male—Stage 1	64	56	17	22	12
Male—Stage 2	13	22	2	7	14
Male—Stage 3	9	12	5	8	16
Female—Stage 1	49	38	15	12	12
Female—Stage 2	8	12	10	12	13
Female—Stage 3	14	21	5	5	7

Stage 1, no fusion; Stage 2, partial fusion; Stage 3, complete fusion. Chi-square test of independence (sex × manubriosternal stage, overall): $\chi^2 = 2.83$, $p = 0.24$.

Table S2. Xiphisternal fusion status by age band and sex, *n*/*N* (% fused).

**Sex**	**30–40 years**	**40–50 years**	**50–55 years**	**55–60 years**	**60–65 years**
Male	33/86 (38.4)	57/90 (63.3)	15/24 (62.5)	30/37 (81.1)	34/42 (81.0)
Female	26/71 (36.6)	36/71 (50.7)	22/30 (73.3)	21/29 (72.4)	22/32 (68.8)

Chi-square test of independence (sex × xiphisternal fusion status, overall; Yates' continuity correction): $\chi^2 = 1.68$, $p = 0.20$. Logistic regression of fusion status on age and sex: **OR for males vs. females** = 1.32, 95% CI 0.91–1.92, $p = 0.14$.

Table S3. Sensitivity analysis with equal-width 10-year age bands: manubriosternal fusion stage and xiphisternal fusion status by age band, *n* (%).

**Stage/status**	**30–40 years ($n = 157$)**	**40–50 years ($n = 161$)**	**50–60 years ($n = 120$)**	**60–65 years ($n = 74$)**
**MS Stage 1 (no fusion)**	**113 (72.0)**	**94 (58.4)**	**66 (55.0)**	**24 (32.4)**
**MS Stage 2 (partial fusion)**	**21 (13.4)**	**34 (21.1)**	**31 (25.8)**	**27 (36.5)**
**MS Stage 3 (complete fusion)**	**23 (14.6)**	**33 (20.5)**	**23 (19.2)**	**23 (31.1)**
**MS Stage 3, % (95% CI)**	**14.6 (10.0–21.0)**	**20.5 (15.0–27.4)**	**19.2 (13.1–27.1)**	**31.1 (21.7–42.3)**
**Non-fused XS (SK−)**	**98 (62.4)**	**68 (42.2)**	**32 (26.7)**	**18 (24.3)**
**Fused XS (SK+)**	**59 (37.6)**	**93 (57.8)**	**88 (73.3)**	**56 (75.7)**
**SK+, % (95% CI)**	**37.6 (30.4–45.4)**	**57.8 (50.0–65.1)**	**73.3 (64.8–80.4)**	**75.7 (64.8–84.0)**

MS, manubriosternal junction; XS, xiphisternal junction; SK−/SK+, non-fused/fused xiphisternal junction. The 50–60-year band combines the 50–55- and 55–60-year bands of the main analysis; the 60–65-year band is limited by the upper age bound of the sample. Manubriosternal stage × age band: $\chi^2 = 33.87$, df = 6, $p < 0.001$; ordered trend for complete fusion (logistic regression on ordinal age-band score): $\beta = 0.269$, $p = 0.011$. Xiphisternal status × age band: $\chi^2 = 47.89$, df = 3, $p < 0.001$; ordered trend: $\beta = 0.621$, $p < 0.001$. 95% CIs are Wilson score intervals.
